# Algorithmic Discovery of Methylation “Hot Spots” in DNA from Lymphoma Patients

**DOI:** 10.4137/cin.s921

**Published:** 2008-09-04

**Authors:** Chris Papageorgio, Robert Harrison, Farahnaz B. Rahmatpanah, Kristen Taylor, Wade Davis, Charles W. Caldwell

**Affiliations:** 1 Department of Internal Medicine/Division of Hematology Oncology, Ellis Fischel Cancer Center, University of Missouri School of Medicine, Columbia, MO 65203, U.S.A; 2 Department of Computer Science, P.O. Box 3994, Atlanta GA 30302, U.S.A; 3 Department of Pathology and Anatomical Sciences, Ellis Fischel Cancer Center, University of Missouri School of Medicine, Columbia, MO 65203, U.S.A; 4 Department of Medical Research, University of Missouri School of Medicine, Columbia, MO 65203, U.S.A

## Abstract

The computational aspects of the problem in this paper involve, firstly, selective mapping of methylated DNA clones according to methylation level and, secondly, extracting motif information from all the mapped elements in the absence of prior probability distribution. Our novel implementation of algorithms to map and maximize expectation in this setting has generated data that appear to be distinct for each lymphoma subtype examined. A “clone” represents a polymerase chain reaction (PCR) product (on average ~500 bp) which belongs to a microarray of 8544 such sequences preserving CpG-rich islands (CGIs) [1]. Accumulating evidence indicates that cancers including lymphomas demonstrate hypermethylation of CGIs “silencing” an increasing number of tumor suppressor (TS) genes which can lead to tumorigenesis.

## Introduction

Non-Hodgkin’s lymphoma (NHL) is a group of malignancies of the immune system with variable clinical behaviors and diverse molecular features. Despite the progress made in classification of NH–ed on classical methods, molecular classifications are a work in progress. Accumulating evidence indicates that hypermethylation of CGIs appears to be a prevalent molecular event in human cancers including NHLs affecting an increasing number of genes. In order to better understand this widespread phenomenon, our laboratory utilized a microarray containing 8544 sequenced CGI clones (on average ≈500 bp in length each) to survey the genome-wide methylation present in low-grade NHLs namely chronic lymphocytic leukemia (CLL), follicular lymphoma (FL) and mantle cell lymphoma (collectively termed small B-cell lymphomas or SBCLs). The data generated in our laboratory by Rahmatpanah F.B. et al. [2] revealed that there was diversity in DNA methylation among different SBCL subtypes and some genes were preferentially methylated in a subtype-related manner. Therefore, we decided to gather all experimentally verified methylated candidate loci in lymphoma patients in order to map them according to their corresponding chromosomal locations. These locations were described by Heisler L.E. et al. [3] who sequenced the complete set of 20,736 clones (9K Subset + 12K Subset) derived from a physical CGI library prepared by others previously [1, 4–6].

Toward this goal, we designed an algorithm to parse and mine these data in order to examine the “randomness” of those differentially methylated candidate loci across the chromosomal landscape. Our aim was to examine computationally the “randomness” of those differentially methylated candidate loci across the chromosomal landscape and relate any apparent non-random DNA methylation “hot spots” on specific chromosomes to potential recurring gene motifs.

### Physical mapping

For the computational aspect of this project involving extraction and reporting of information we decided to use Perl language because it is well known as the main language for report generation and ad hoc data mining [7]. When writing the code, we took advantage of Perl’s feature that allowed one to use “hard” references to any piece of data or code. Indeed, any scalar may hold a hard reference. Because arrays and hashes contain scalars, one can easily build arrays of arrays, arrays of hashes, hashes of arrays, arrays of hashes of functions, and so on. We decided to use references to variables because of the rather complex data structures that had to be parsed and combined.

For data reading and manipulation in Microsoft Excel format, the “Comma Separated Values” (CSV) file format is still widely used because it has a better performance under compression. For this reason all spreadsheets in Excel were saved as CSV files. Therefore, in order to combine fields of data in CSV string and parse CSV strings into a workable array, Text:CSV module (version 0.5) was utilized (parsing with this module can be done in simple array mode, returning a reference to an array if the columns are not named).

In order to extract from the data only those differentially methylated candidate loci having the highest methylation scores by differential methylation hybridization, we defined a subroutine which returned an array of references to an array of the extracted columns. Strict pragma and lexical variables were used both in the two subroutines and the main body of the program. The ouput was printed in the terminal in text format as groups of identified methylated loci per chromosome each recognized by its name and specific chromosomal location.

In order to develop a chromosomal ideogram, the MapChart software was downloaded from the website at http://www.joinmap.nl [8]. Robust non-parametric test for chromosome-wide clustering was applied as described previously [2].

### Motif extraction

Motifs can be used as “generalized sequences” [9, 10] which we can assume to have common functionality, e.g. transcription start sites (TSS), by deduction through sequence conservation. This is the reason why we decided to proceed with a sequence-driven algorithm in order to identify possible candidate regulatory elements involved, perhaps, in DNA methylation of our genes. The central computational aspect of the problem is extracting motif information in the absence of a prior probability distribution. Therefore straightforward expectation maximization algorithms of heuristic techniques such as MEME or AlignAce, which aid in identifying common motifs in sets of unaligned sequences [11, 12] are not directly applicable. Instead it is necessary to use a machine learning approximation to expectation maximization such as K-means, K-nn or C-means clustering algorithms. This approach has the attractive property that it assumes nothing about the distribution of base frequencies and derives the distribution from the input sequence data. This approach is similar to using a self-organizing map [12a]. Implemented properly, these algorithms can extract features that are in common among a set of objects as well as probability and likelihood information over the set of objects.

Motifs were found using an iterative censored fuzzy C-means algorithm. Sets of sequences drawn from a given disease class were iteratively compared with each other, and those areas where significant alignments could be identified were then used to update the sequence probability profiles. The sequence alignments from both possible strands were used since either strand of the sequence could have a significant alignment. When no alignment could be found, then the sequence probability profile was moved towards a uniform distribution. This process was repeated until a stationary state was reached.

After convergence, in addition to reporting the profile information, the entropy of the profile relative to a uniform distribution was reported. Maxima of the entropy correspond to regions where the profile is least uniform and therefore has the most signal. These regions were extracted and then examined to see if they were the same in different disease classes (see supplementary information).

## Implementation

Using an algorithmic approach and stricter criteria than in earlier work from our laboratory [13] (>1.5-fold difference between tumor and normal tissue; present in >25% of patient samples) two aberrantly methylated loci were observed in CLL, 95 in FL 49 in MCL. Each one of these selected methylated loci or clones was mapped to its genomic location using MapChart^©^ software. By using a robust nonparametric statistical test for chromosome-wide methylation localization and clustering that takes all loci into consideration, we detected statistically significant clustering of aberrantly methylated clones on chromosome 2 (p = 0.0005), 9 (p = 0.0374) and 19 (p = 0.0078) in FL only. Using an iterative censored fuzzy C-means algorithm we found a set of motifs that recur throughout a disease type of the methylated loci. We hypothesize that these sites could be transcription factor binding sites acknowledging that the probability of a site being a functional site is substantially increased when an annotated transcription start-site (TSS) is nearby as cataloged in the Eukaryotic Promoter Database. The computational aspects of the problem in this paper involve, firstly, selective mapping of methylated DNA clones according to methylation level and, secondly, extracting motif information from all the mapped elements in the absence of prior probability distribution. Our novel implementation of algorithms to map and maximize expectation in this setting has generated data that appear to be distinct for each disease type examined.

**Figure 1:**
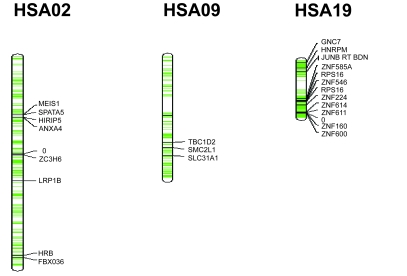
Ideogram depicting the “hot spots” discovered on chromosomes 2, 9 and 19 that demonstrated statistical significance (loci marked “0” denote genes that have not been investigated yet for identity).

The 146 differentially methylated candidate loci were mapped to their corresponding chromosomal locations to examine their “randomness” across the chromosomal landscape (Fig. 1).

This process revealed interesting observations. Firstly, that the allocations of these clones are well distributed across all chromosomes, albeit with sparse representation on certain regions known to be minimally methylated at a genomic level [14, 15]. Secondly, the distribution of these hypermethylated loci does not appear to be evenly spread across all chromosomal sites. In the present study, chromosomes 2 (p = 0.0005), 9 (p = 0.0374) and 19 (p = 0.0078) have a statistically significant over-representation of methylated loci in only one subtype of our lymphoma patients (see supplementary information).

A set of motifs that recur throughout a disease type of the methylated sites was derived by this process. We hypothesize that these sites could be transcription factor binding sites acknowledging that the probability of a site being a functional site is substantially increased when an annotated TSS is nearby as cataloged in the Eukaryotic Promoter Database (see supplementary information).

## Discussion

Most recent work in our laboratory by Taylor K.H. et al. [13] revealed that chromosome 19 has an over-representation of methylated loci in a large-scale analysis of acute lymphoblastic leukemia (ALL) patient samples. This, again, is in accordance with our present demonstration of chromosome 19 being “burdened” by clusters of methylated loci in FL patients in a statistically significant way. It is interesting to note that chromosome 19 has the highest gene density of all human chromosomes, more than double the genome-wide average. The large clustered gene families, corresponding to high G+C content, CGI and density of repetitive DNA indicate a chromosome rich in biological and evolutionary significance. By comparison, the observed clusters of methylated loci in both ALL and FL encompass a region on chromosome 19 which is believed to harbor TS genes associated with many types of cancer including breast, lung, colorectal, ovarian and brain. The algorithmic discovery in our study of a common motif among methylated loci provides a plausible answer to the question about a seemingly targeted initiation of methylation that can lead to TS genes silencing and human cancer.

Our hypothesis is that a common motif shared by the aberrantly methylated loci may be a DNA binding site for transcription factors that promote aberrant methylation and thus what appears to be a non-random targeted methylation. In this way, chromosome 19 may play a key role in, facilititating the initiation and spreading of methylation and TS genes silencing leading to neoplastic transformation. Hence, our computational approach strengthens and expands further the data generated by Taylor et al. as it first confirms the importance of chromosome 19 in a different lymphoblastic disease like FL and then provides a mechanism for possible chromosome-specific preferential DNA methylation. Data like these can be examined not only as a means for early diagnosis of cancer but also as biomarkers for targeted therapies and monitoring of response to treatment.

This brief report demonstrates the power of computational and statistical approach to high-throughput CGI microarray data in cancer research. The fact that this particular data analysis is stemming from a study of ~50% of potential CGI sequences in the human genome does not diminish the value of discovering chromosomal clusters of methylation within lymphoma subclasses. By integrating our quantitative microarray data through computational and statistical models we aspire to determine sites or “hot spots” within the human genome at which we could intervene therapeutically at a genetic or epigenetic level.

Any apparent non-random DNA methylation “hot spots” on specific chromosomes associated with specific NHL subtypes should warrant not only further studies of the chromatin landscape but also clinical correlations prospectively or retrospectively with regard to subtype-related diagnosis, treatment outcome and prognosis. From a cancer treatment standpoint, the effect of methylation on silencing tumor-supprressor genes is important because it is now possible to reverse these changes with DNA methylation inhibitors, thereby restoring the tumor-suppressor gene function. To avoid misinterpretation of methylation data for a single gene, methods like ours that comprehensively analyze DNA methylation data at the whole-genome level become of utmost importance as they help to develop and refine epigenetic therapies for cancer.

## Supplementary Material
